# Blood neutrophil extracellular traps: a novel target for the assessment of mammary health in transition dairy cows

**DOI:** 10.1186/s40104-022-00782-4

**Published:** 2022-11-16

**Authors:** Luyi Jiang, Huizeng Sun, Fengfei Gu, Jin He, Fengqi Zhao, Jianxin Liu

**Affiliations:** 1 Institute of Dairy Science, College of Animal Sciences, Zhejiang University, Hangzhou, China; 2 Ministry of Education Key Laboratory of Molecular Animal Nutrition, Zhejiang University, Hangzhou, China; 3 Institute of Animal Genetics and Reproduction, College of Animal Sciences, Zhejiang University, Hangzhou, China; 4 Department of Animal & Veterinary Sciences, University of Vermont, Burlington, USA

**Keywords:** Blood-milk barrier, Differential somatic cell count, Mastitis risk, Neutrophil extracellular traps

## Abstract

**Background:**

Mammary health is important for transition dairy cows and has been well recognized to exert decisive effects on animal welfare. However, the factors influencing mammary health are still unclear. Differential somatic cell count (DSCC) could reflect the mastitis risk since it is the percentage of neutrophils plus lymphocytes in total somatic cells and could be reflective of mammary health of dairy cows. This work aimed to investigate the assessment and prognosis of the health of transition cows based on blood neutrophil extracellular traps (NETs).

**Results:**

Eighty-four transition Holstein dairy cows were selected. The serum was sampled in all the animals at week 1 pre- and postpartum, and milk was sampled at week 1 postpartum. Based on the DSCC in milk at week 1, cows with lower (7.4% ± 4.07%, *n* = 15) and higher (83.3% ± 1.21%, *n* = 15) DSCCs were selected. High DSCC cows had higher levels of red blood cell counts (*P* < 0.05), hemoglobin (*P* = 0.07), and hematocrit (*P* = 0.05), higher concentrations of serum oxidative variables [(reactive oxygen species (*P* < 0.05), malondialdehyde (*P* < 0.05), protein carbonyl (*P* < 0.05), and 8-hydroxy-2-deoxyguanosine (*P* = 0.07)], higher levels of serum and milk NETs (*P* < 0.05) and blood-milk barrier indicators, including serum *β*-casein (*P* = 0.05) and milk immunoglobulin G2 (*P* = 0.09), than those of low DSCC cows. In addition, lower concentrations of serum nutrient metabolites (cholesterol and albumin) (*P* < 0.05) and a lower level of serum deoxyribonuclease I (*P* = 0.09) were observed in high DSCC cows than in low DSCC cows. Among the assessments performed using levels of the three prepartum serum parameters (NETs, deoxyribonuclease I and *β*-casein), the area under the curve (0.973) of NETs was the highest. In addition, the sensitivity (1.00) and specificity (0.93) were observed for the discrimination of these cows using NETs levels with a critical value of 32.2 ng/mL (*P* < 0.05).

**Conclusions:**

The formation of NETs in blood in transition dairy cows may damage the integrity of the blood-milk barrier and thereby increase the risk for mastitis in postpartum cows.

## Introduction

Mastitis is a major health problem in transition dairy cows, causing considerable economic losses in the dairy industry [1]. To date, the milk somatic cell count (SCC) is broadly considered a valuable biomarker for monitoring the inflammatory response to mastitis worldwide. In addition to mammary epithelial cells, the somatic cells present in milk also consist of white blood cells, including neutrophils, lymphocytes, monocytes, and macrophages. Among them, neutrophils are innate immune phagocytes that exert crucial roles in immune defense [2], while lymphocytes play an important role in the induction and suppression of immune responses [3, 4]. These immune cells are crucial in the inflammatory responses of the mammary gland, and their counts are important indicators of the mastitis risk in dairy cows [5]. Hence, differentiation of immune cell in milk is helpful for description of the mammary health of dairy cows. The differential somatic cell count (DSCC), expressed as the percentage of neutrophils plus lymphocytes in total SCC, could be better reflective of the mammary health in dairy cows. However, the DSCC has low stability in low SCC samples, thus combination of DSCC and SCC is sometimes used to identify the mammary inflammation in practical application [5, 6].

Leukocytes consist mostly of lymphocytes and macrophages in the uninfected mammary gland [7]. In cows with mastitis, however, the percentage of neutrophils in milk can be increased to 75%, indicating that mastitis risk is closely related to increased neutrophil to total leukocyte ratios [7]. The anti-inflammatory activity of neutrophils is mediated by three mechanisms, i.e., phagocytosis, degranulation, and the release of neutrophil extracellular traps (NETs). Among them, the formation of NETs is dependent on the production of reactive oxygen species (ROS). The NETs are large, extracellular, and web-like structures comprising cytosolic and granule proteins that are assembled on a scaffold of decondensed chromatin [8]. The NETs formation is a novel microbe-eliminating process of neutrophils and has received much attention recently. However, if dysregulated, NETs can damage organ tissues, contributing to the pathogenesis of immune-related diseases [9, 10]. The NETosis-derived products exert diagnostic potential for diseases related to inflammation [11]. In our previous work, NETs could be used as a potential indicator for the prognosis of mastitis risk in transition cows [12].

The intact blood-milk barrier prevents the uncontrolled exchange of soluble and cellular components between the blood and milk in the mammary gland [13]. The barrier is formed by endothelial cells, connective tissue, the basal membrane, and epithelial cells. Among these, epithelial cells are the main cell type that provides the semipermeability of this barrier, allowing only the transfer of the components required for milk production, and this barrier is necessary for maintaining the mammary homeostasis [13]. In transition dairy cows, the permeability of tight junctions is prone to increase, leading to a breakdown of the blood-milk barrier and an increase in mastitis risk [14].

For cow’s health, the transition period is a great physiological challenge. Our previous study suggested that the formation of blood NETs in transition dairy cows can increase the risk of postpartum mastitis [12]. Therefore, we hypothesized that the formation of blood NETs is different between transition dairy cows with low DSCC and those with high DSCC, and the NETs are associated with changes in the permeability of the blood-milk barrier and can be used as an indicator prepartum to estimate the risk of mastitis postpartum.

## Materials and methods

This study was conducted in accordance with the guidelines for animal welfare and the experimental protocols for animal care, approved by the Animal Care Committee of Zhejiang University (Hangzhou, Zhejiang, China).

### Animals and management

Ninety-three multiparous healthy Holstein dairy cows at week 1 prepartum were collected, fed, and managed at a local dairy farm. Nine cows were then removed due to their health problems such as clinical mastitis, ketosis, calving disorders (twins), and metritis during the experiment. As a result, 84 multiparous healthy Holstein dairy cows were selected for the current study. All the selected cows were of similar parity (2.7 ± 0.09) and were monitored for health weekly with no apparent disease symptoms occurring during the study. Briefly, cows were monitored daily for mammary appearance, gait, appetite, general appearance, alertness, and vaginal discharge from week 1 prepartum to week 1 postpartum according to a previously described method [15]. Diagnosis and treatment of diseases were conducted weekly by the farm veterinarian. The serum was sampled at week –1 and + 1 related to calving, and milk was sampled at week 1 postpartum. Throughout the whole period, dairy cows were housed in individual tie stalls, were bedded with sawdust, were milked three times daily, and were given free access to water. Diets were provided as total mixed rations three times daily at 06:30, 13:00, and 18:30 to allow approximately 5% orts.

### Sample collections

Blood samples were collected from the coccygeal vein (09:30 to 10:00). One portion of the blood was collected into 2-mL EDTA anticoagulant vacutainer tubes for analysis of the hematological parameters. Another blood sample was collected into 10-mL pro-coagulation tubes and kept at 4 °C. The sample was then centrifuged at 3000 × *g* for 15 min to collect serum samples, which were stored at – 80 °C until analysis.

Milk samples were obtained at the three milking times by using composite milk samplers (Waikato Milking Systems NZ Ltd., Hamilton, New Zealand) in a proportion of 4:3:3. One milk sample was stored with added bronopol tablets for SCC and DSCC analysis using a CombiFoss 7 DC (FOSS Analytical, Hillerød, Denmark), and another milk sample was stored at – 80 °C until analysis.

**Fig. 1:**
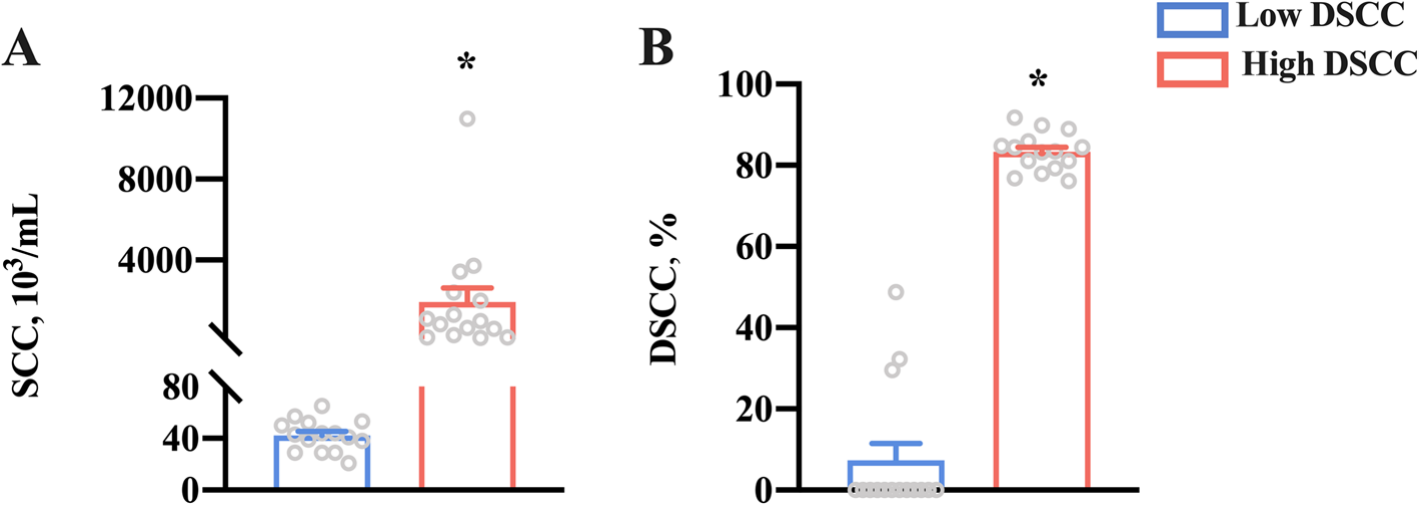
Somatic cell count (SCC, **A**) and differential SCC (DSCC, **B**) in dairy cows with low- and high-DSCC at week + 1 relative to calving. *n* = 15. Error bars indicate the standard error of the mean. *P < 0.05

With a power of 0.999 for DSCC, 15 dairy cows with lower DSCC (7.4% ± 4.07%) and higher DSCC (83.3% ± 1.21%) values were selected and defined as low DSCC and high DSCC cows, respectively (Fig. 1). The remaining 54 cows were excluded from the analysis of identifying factors of mastitis risk in cows, but all the data of 84 cows were used to analyze the correlations among DSCC, NETs-related indices, and blood-milk barrier-related indices.

### Measurement of blood and serum parameters

#### Hematological parameters

Blood samples were used for measurements of the levels of hematological parameters, including white blood cell, lymphocyte, monocyte, neutrophil, eosinophil, basophil, platelet, red blood cell, hemoglobin, and hematocrit, using an automatic blood cell analyzer URIT-5181 (Guilin Youlite Medical Electronics Co., Ltd., Guilin, China).

#### Serum biochemical parameters

The levels of the serum biochemical parameters, including glucose, triglyceride, nonesterified fatty acid, *β*-hydroxybutyrate, cholesterol, alanine aminotransferase, aspartate aminotransferase, total protein, albumin, blood urea nitrogen, and creatinine, were analyzed with colorimetric commercial kits (Ningbo Medical System Biotechnology Co., Ltd., Ningbo, China) using an Auto Analyzer 7020 instrument (Hitachi High-Technologies Corp., Tokyo, Japan).

#### Serum oxidative damage levels

Serum ROS concentration was analyzed using the ROS kit (#E004-1–1) with a fluorescent dichlorofluorescein-diacetate (DCFH-DA) probe following the manufacturer’s instructions (Nanjing Jiancheng Bioengineering Institute, Nanjing, China). Serum lipid peroxidation levels were measured with a malondialdehyde (MDA) assay kit (#A003-1–2, Nanjing Jiancheng Bioengineering Institute). Measurement of the serum protein oxidation level as the concentration of protein carbonyls (PC) was carried out with a PC kit (#A087-1–2, Nanjing Jiancheng Bioengineering Institute) following the manufacturer’s instructions. In addition, the 8-hydroxy-2-deoxyguanosine (8-OHdG) concentration was measured with an 8-OHdG kit (#E-EL-0028c, Elabscience Biotechnology, Wuhan, China) according to the method provided by the manufacturer’s instructions.

### Measurement of serum and milk NETs-related indices

The blood samples were clotted in serum tubes for 2 h at 4 °C and then centrifuged at 1000 × *g* for 20 min. The milk samples were collected after centrifuging the milk at 2000 × *g* for 15 min at 4 °C. Freshly prepared samples of serum and milk were used for the measurements of the NETs level [16]. Briefly, the antibodies [anti-myeloperoxidase (MPO), 1:2000 dilution in sterile PBS] were captured onto 96-well high-binding capacity ELISA microplates and incubated overnight at 4 °C. The plates were washed with PBS-Tween 20 twice, blocked with 5% bovine serum albumin (BSA, 200 μL/well), incubated at 4 °C for another 2 h, and then washed with PBS-Tween 20 twice again. Subsequently, the fresh serum and milk samples were incubated in ELISA plates overnight at 4 °C. The plates were washed with PBS-Tween 20 twice, and 50 μL of the detection antibody (HRP-labeled anti-dsDNA, 1:500, mouse) was added to each well and incubated for another 1 h at 4 °C in the dark. The plates were then washed again, 50 μL of TMB peroxidase substrate was added to each well and incubated for 30 min, and the reaction was terminated by the addition of HCl (1 mol/L, 50 μL/well). The absorbance was determined using a microplate photometer (Thermo Fisher Scientific, Tokyo, Japan) at 450 nm.

The concentrations of MPO (#A044-1–1) in serum and milk samples were measured using an MPO kit (Nanjing Jiancheng Bioengineering Institute). The contents of neutrophil elastase (NE, #CK-EN78022) and deoxyribonuclease I (DNase I, #CK-EN77261) in serum and milk samples were determined using corresponding kits (Quanzhou Ruixin Science & Technology, Quanzhou, China).

### Measurements of blood-milk barrier-related indices in serum and milk

As the indicators of blood-milk barrier integrity, the serum concentration of lactose (#K-LOLAC) was determined by a kit (Megazyme International, Bray, Ireland) and the serum concentrations of *α*-lactalbumin (#CK-EN77481) and *β*-casein (#CK-EN77015) were also measured using corresponding kits (Nanjing Jiancheng Bioengineering Institute and Quanzhou Ruixin Science & Technology).

Additionally, as indicators of blood-milk barrier integrity, the milk concentrations of Na^+^/K^+^ (#C002-1–1 and #C001-2–1), BSA (#A028-1–1), immunoglobulin G1 (IgG1, #CK-EN77908), and immunoglobulin G2 (IgG2, #CK-EN77909) were determined using corresponding kits (Nanjing Jiancheng Bioengineering Institute and Quanzhou Ruixin Science & Technology).

### Statistical analysis

Correlation of DSCC, neutrophil counts, NETs-related parameters, and blood-milk barrier-related indices for 84 dairy cows were analyzed using an R package (ggcorrplot, v. 0.1.3). The correlation was then classified as negative (*r* = – 1.00 to – 0.30), negligible (*r* = – 0.31 to 0.30), or positive (*r* = 0.31 to 1.00), according to the method described by Bikker et al. [17] and Mukaka [18]. The statistical significance was declared at FDR < 0.05.

To identify factors of mastitis risk in cows, the data between low and high DSCC were compared using a PROC MIXED model with health status (Hs), sampling week (Wk), and interaction (Hs × Wk) as fixed effects and dairy cows as random effects. The data are expressed as the mean ± standard error of the mean (SEM) and were analyzed using Statistical Analysis System software (version 9.1; SAS Institute, Inc., Cary, NC, USA). The statistical significance was declared at *P* < 0.05, and trends were indicated at 0.05 ≤ *P* < 0.10.

The prognostic potential of serum NETs, DNase I, and *β*-casein measured in the prepartum period for postpartum mastitis risk was assessed using receiver operator characteristic (ROC) curves. The generation of the ROC curves was performed using IBM SPSS Statistics 23.0 software (International Business Machines Corp., Armonk, New York 10,504, USA). The area under the curve (AUC) was calculated to evaluate the diagnostic accuracy and to compare the AUCs of all tested parameters separately. An AUC of 0.5 suggests no discrimination, 0.7 to 0.8 is considered acceptable, 0.8 to 0.9 is considered excellent, and an AUC higher than 0.9 is considered outstanding.

## Results

### Correlation of DSCC, NETs, and blood-milk barrier in transition dairy cows

**Fig. 2:**
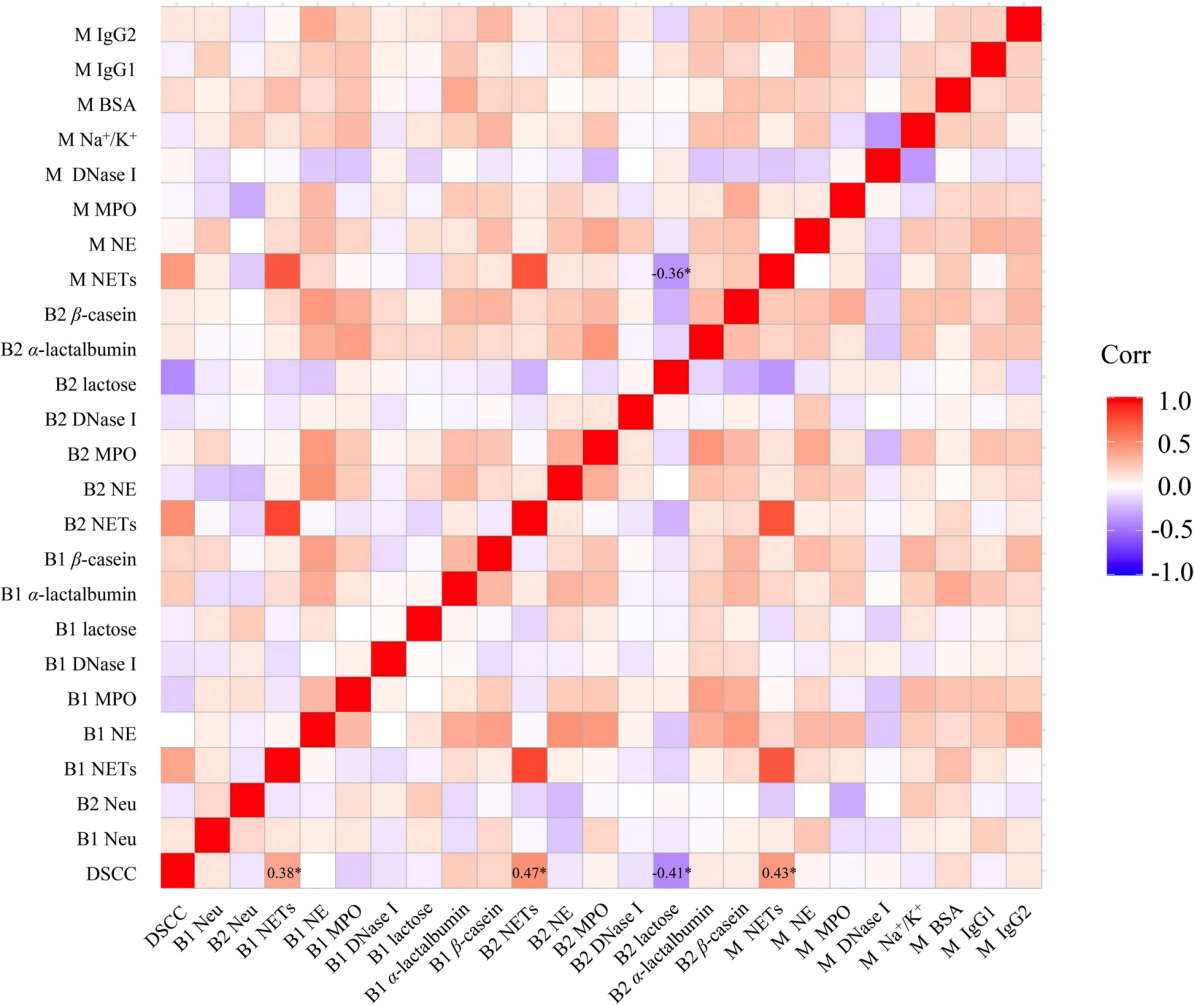
Correlation (Corr) analyses of differential somatic cell count (DSCC), neutrophil counts, NETs-related indices, and blood-milk barrier-related indices at week – 1 (prepartum) or + 1 (postpartum) relative to calving in dairy cows. *n* = 84. * FDR < 0.05. B1: Prepartum serum; B2: Postpartum serum; M: Milk; NETs: Neutrophil extracellular traps; NE: Neutrophil elastase; MPO: Myeloperoxidase; Na^+^/K^+^: Ratio of sodium to potassium; BSA: Bovine serum albumin; IgG: Immunoglobulin G

The results of the correlation analyses of DSCC, neutrophil counts, NETs-related indices, and blood-milk barrier-related indices at week – 1 (prepartum) or + 1 (postpartum) relative to calving are shown in Fig. 2. Positive correlations were found between DSCC and the level of serum NETs at week 1 prepartum (*r* = 0.38, FDR < 0.01), the level of serum NETs at week 1 postpartum (*r* = 0.47, FDR < 0.01), and the level of milk NETs at week 1 postpartum (*r* = 0.43, FDR < 0.01). A negative correlation was observed between DSCC and serum lactose level at week 1 postpartum (*r* = – 0.41, FDR < 0.01), with negligible correlations between the DSCC and the levels of the other indices. In addition, the level of milk NETs at week 1 postpartum was negatively correlated with the serum lactose level at week 1 postpartum (*r* = – 0.36, FDR < 0.01), with negligible correlations between the levels of NETs and blood-milk barrier-related indices.

### Health status and nutritional metabolite levels

The hematological parameters in dairy cows with low and high DSCC at week 1 pre- and postpartum are presented in Table 1. Serum levels of hemoglobin (*P* = 0.07) and hematocrit (*P* = 0.05) tended to be higher, and serum level of red blood cell (*P* = 0.03) was higher in cows with high DSCC than in those with low DSCC both pre- and postpartum. No differences were observed (*P* > 0.10) in other hematological parameters between low DSCC and high DSCC cows. Moreover, the number of serum neutrophil tended to be lower (*P* = 0.06) in the postpartum cows than in the prepartum cows. The serum counts of monocyte (*P* = 0.01) and platelet (*P* = 0.03) were higher, and the serum count of eosinophil (*P* < 0.01) was lower in the postpartum cows than in the prepartum animals. No differences were found (*P* > 0.10) in the levels of other hematological parameters between prepartum and postpartum dairy cows. There existed an interaction (Hs × Wk) trend for effect on eosinophil (*P* = 0.05) and significant interaction effects on white blood cell (*P* = 0.02) and neutrophil (*P* = 0.03). Furthermore, cows with high DSCC in postpartum had lower (*P* < 0.05) counts of white blood cell, neutrophil, and eosinophil relative to those in prepartum.

**Table 1:** Hematological parameters in dairy cows with low- and high-DSCC at week –1 or + 1 relative to calving^1^

Items	– 1 Wk^2^		+ 1 Wk^2^		SEM	*P*-value^3^		
	Low DSCC	High DSCC	Low DSCC	High DSCC		Hs	Wk	Hs × Wk
White blood cell, 10^9^/L	11.7^ac^	12.3^a^	12.3^ac^	9.6^bc^	0.64	0.54	0.11	0.02
Lymphocyte, 10^9^/L	5.94	5.40	6.09	5.12	0.508	0.60	0.86	0.53
Monocyte, 10^9^/L	1.03^b^	1.05^b^	1.39^a^	1.15^ab^	0.058	0.41	0.01	0.16
Neutrophil, 10^9^/L	4.53^ac^	5.48^a^	4.71^ac^	3.19^bc^	0.287	0.62	0.06	0.03
Eosinophil, 10^7^/L	18.5^ab^	37.7^a^	9.8^b^	11.2^b^	4.07	0.32	< 0.01	0.05
Basophil, 10^7^/L	2.47	2.67	3.09	2.24	0.212	0.50	0.80	0.16
Platelet, 10^9^/L	314^ab^	309^b^	349^ab^	389^a^	14.8	0.61	0.03	0.37
Red blood cell, 10^12^/L	6.29^b^	7.08^a^	6.43^ab^	6.85^ab^	0.134	0.03	0.86	0.46
Hemoglobin, g/L	107^b^	120^a^	110^ab^	115^ab^	2.2	0.07	0.91	0.33
Hematocrit, %	30.3	34.0	31.0	32.8	0.65	0.05	0.84	0.47

^1^The data are expressed as the mean ± SEM, *n* = 15. ^a,b,c^*P* < 0.05

^2^*DSCC* Differential somatic cell count, *– 1 Wk* Week – 1 relative to calving, + *1 Wk* Week + 1 relative to calving

^3^*Hs* Health status, *Wk* Sampling week, *Hs* × *Wk* Interaction between health status and sampling week

Serum nutrient metabolite levels in dairy cows with low and high DSCC at week 1 pre- and postpartum are shown in Table 2. The concentrations of serum cholesterol (*P* = 0.03) and albumin (*P* = 0.03) were lower in high DSCC cows than in low DSCC cows. No significant differences were found (*P* > 0.05) in the levels of other metabolites between the two groups of cows. Serum concentrations of nonesterified fatty acid, *β*-hydroxybutyrate, and aspartate aminotransferase were higher (*P* < 0.01), whereas glucose, triglyceride, cholesterol, and creatinine concentrations were lower (*P* < 0.01) in the postpartum cows than in the prepartum cows, with no differences (*P* > 0.05) in the levels of other metabolites between prepartum and postpartum dairy cows. There was an interaction (Hs × Wk) trend for effect on ALT (*P* = 0.07).

**Table 2:** Serum nutrient metabolites in dairy cows with low- and high-DSCC at week – 1 or + 1 relative to calving^1^

Items^2^	– 1 Wk^3^		+ 1 Wk^3^		SEM	*P*-value^4^		
	Low DSCC	High DSCC	Low DSCC	High DSCC		Hs	Wk	Hs × Wk
Glucose, mmol/L	3.31^a^	3.35^a^	2.80^b^	3.10^ab^	0.062	0.19	< 0.01	0.20
Triglyceride, μmol/L	223^a^	231^a^	90^b^	95^b^	10.1	0.52	< 0.01	0.90
NEFA, μmol/L	271^b^	246^b^	791^a^	845^a^	61.1	0.89	< 0.01	0.68
BHBA, μmol/L	467^b^	458^b^	931^a^	743^a^	46.1	0.24	< 0.01	0.24
Cholesterol, mmol/L	3.01^a^	2.69^ac^	2.56^bc^	2.22^b^	0.071	0.03	< 0.01	0.90
ALT, U/L	19.9	21.5	20.8	20.2	0.48	0.67	0.78	0.07
AST, U/L	64^b^	69^b^	113^a^	101^a^	4.15	0.59	< 0.01	0.18
Total protein, g/L	68.9	70.3	69.2	71.3	0.85	0.40	0.64	0.82
Albumin, g/L	33.8^a^	32.3^ab^	33.5^a^	31.6^b^	0.31	0.03	0.32	0.63
Blood urea nitrogen, mmol/L	4.49	4.20	4.54	3.66	0.143	0.10	0.21	0.15
Creatinine, μmol/L	95.4^a^	90.1^ab^	86.3^b^	79.8^bc^	2.01	0.25	< 0.01	0.77

^1^The data are expressed as the mean ± SEM, *n* = 15. ^a,b,c^*P* < 0.05

^2^*NEFA* Nonesterified fatty acid, *BHBA β*-hydroxybutyrate, *ALT* Alanine aminotransferase, *AST* Aspartate aminotransferase

^3^*DSCC* Differential somatic cell count, *– 1 Wk* Week – 1 relative to calving, + *1 Wk* Week + 1 relative to calving

^4^*Hs* Health status, *Wk* Sampling week, *Hs* × *Wk* Interaction between health status and sampling week

### Oxidative damage

**Fig. 3:**
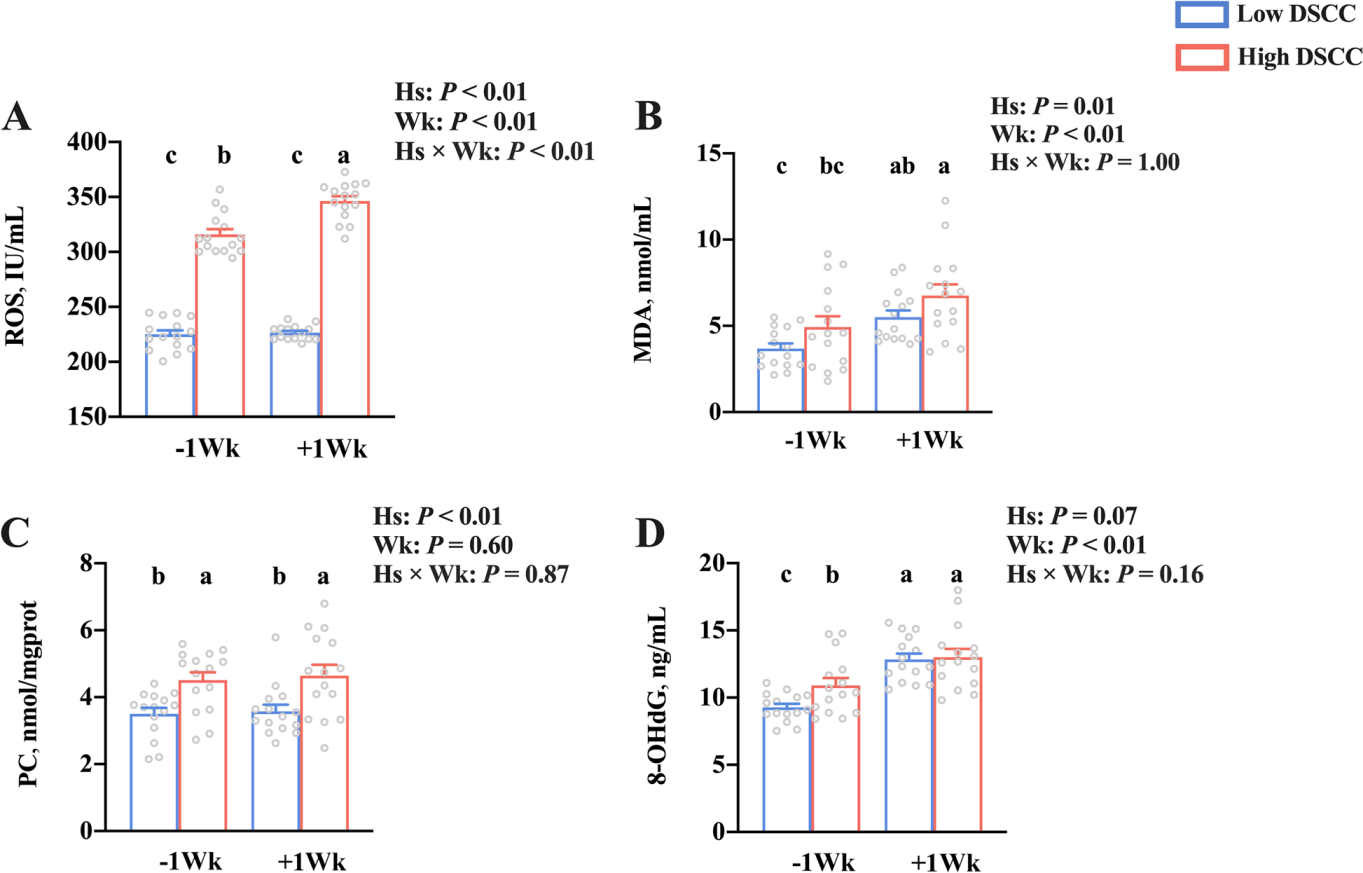
Serum oxidative damage in dairy cows with low- and high-DSCC at week – 1 or + 1 relative to calving. **A** Reactive oxygen species (ROS), **B** malondialdehyde (MDA), **C** protein carbonyl (PC), and **D** 8-hydroxy-2 deoxyguanosine (8-OHDG). *n* = 15. Error bars indicate the standard error of the mean. ^a,^^b,^^c^*P* < 0.05. DSCC: Differential somatic cell counts; Hs: Health status; Wk: Sampling week; Hs × Wk: Interaction between health status and sampling week

Serum concentrations of oxidative damage-related parameters in dairy cows with low and high DSCC are shown in Fig. 3. The serum levels of ROS, MDA, and PC were higher (*P* < 0.05) in cows with high DSCC than in cows with low DSCC. The 8-OHdG level tended to be higher (*P* = 0.07) in high DSCC cows than in low DSCC cows. In addition, the serum levels of ROS, MDA, and 8-OHdG were higher (*P* < 0.05) in the postpartum cows than in the prepartum animals, but no significant difference was found (*P* > 0.05) in PC levels between pre- and postpartum dairy cows. The interaction effect (Hs × Wk) was significant for ROS level (*P* < 0.01). Compared to prepartum cows with low DSCC, the level of ROS was higher (*P* < 0.05) in cows with high DSCC in both pre- and postpartum. Dairy cows with high DSCC showed higher ROS level in postpartum than in prepartum (*P* < 0.05).

### Formation of NETs and blood-milk barrier integrity

Serum and milk concentrations of NETs-related indices are presented in Table 3. The concentration of serum NETs was higher (*P* < 0.01) in high DSCC cows than in low DSCC cows. The serum levels of DNase I tended to be lower (*P* = 0.09) in high DSCC cows, with no significant difference (*P* > 0.05) found in NE and MPO levels between low- and high-DSCC cows. The serum NE concentration was higher (*P* < 0.01) in the postpartum period than in the prepartum period. No significant difference (*P* > 0.05) was found in the levels of NETs, MPO, or DNase I between pre- and postpartum dairy cows. The concentration of milk NETs was higher (*P* < 0.01) in high DSCC cows than in low DSCC cows, with no significant difference (*P* > 0.05) in levels of NE, MPO, and DNase I. The interaction effect (Hs × Wk) was not significant (*P* > 0.05) for the levels of any serum NETs-related indices.

**Table 3:** Neutrophil extracellular traps-related indices in serum and milk of dairy cows with low- and high-DSCC at week – 1 or + 1 relative to calving^1^

Items	– 1 Wk^2^		+ 1 Wk^2^			*P*-value^3^		
	Low DSCC	High DSCC	Low DSCC	High DSCC	SEM	Hs	Wk	Hs × Wk
*In serum*								
Neutrophil extracellular traps, ng/mL	26.4^b^	40.3^a^	25.2^b^	41.6^a^	1.16	< 0.01	0.98	0.27
Neutrophil elastase, U/L	110^ab^	117^b^	136^ab^	138^a^	3.7	0.63	< 0.01	0.53
Myeloperoxidase, U/L	230	237	259	275	12.8	0.71	0.11	0.83
Deoxyribonuclease I, U/L	99	94	104	96	2.0	0.09	0.43	0.74
*In milk*								
Neutrophil extracellular traps, ng/mL	-	-	23.3	38.0	1.55	< 0.01	-	-
Neutrophil elastase, U/L	-	-	102	108	4.9	0.56	-	-
Myeloperoxidase, U/L	-	-	362	376	16.3	0.68	-	-
Deoxyribonuclease I, U/L	-	-	85.4	78.9	2.95	0.28	-	-

^1^The data are expressed as the mean ± SEM, *n* = 15. ^a,b,c^*P* < 0.05

^2^*DSCC* Differential somatic cell count, *– 1 Wk* Week – 1 relative to calving, + *1 Wk* Week + 1 relative to calving

^3^*Hs* Health status, *Wk* Sampling week, *Hs* × *Wk* Interaction between health status and sampling week

The serum and milk concentrations of blood-milk barrier integrity indicators are shown in Table 4. The concentration of serum *β*-casein tended to be higher (*P* = 0.05) in the cows with high DSCC than in the cows with low DSCC. No significant difference (*P* > 0.05) was found in serum levels of lactose and *α*-lactalbumin between low and high DSCC cows. The serum concentrations of *α*-lactalbumin (*P* = 0.01) and *β*-casein (*P* = 0.04) were higher, while the lactose concentrations were lower (*P* < 0.01) in the postpartum cows than in the prepartum cows. In addition, the milk concentration of IgG2 tended to be higher (*P* = 0.09) in high DSCC cows than in low DSCC cows, with no significant differences (*P* > 0.05) in the Na^+^/K^+^ ratio, BSA level, or IgG1 level. There was no interaction effect (Hs × Wk) observed (*P* > 0.05) for the concentrations of any blood-milk barrier integrity indicators in serum.

**Table 4:** Blood-milk-barrier integrity indices in serum and milk of dairy cows with low- and high-DSCC at week – 1 or + 1 relative to calving^1^

Items	– 1 Wk^2^		+ 1 Wk^2^			*P*-value^3^		
	Low DSCC	High DSCC	Low DSCC	High DSCC	SEM	Hs	Wk	Hs × Wk
*In serum*								
Lactose, μmol/L	3.51^b^	3.29^b^	7.59^a^	6.69^a^	0.389	0.39	< 0.01	0.57
*α*-lactalbumin, μg/mL	0.83^b^	0.94^ab^	1.03^a^	1.09^a^	0.038	0.28	0.01	0.69
*β*-casein, μg/mL	4.72^b^	5.33^a^	5.12^ab^	5.53^a^	0.108	0.05	0.04	0.48
*In milk*								
Na^+^/K^+^ ratio	-	-	0.250	0.253	0.0098	0.87	-	-
Bovine serum albumin, mg/mL	-	-	0.689	0.717	0.0185	0.46	-	-
Immunoglobulin G1, μg/mL	-	-	36.3	37.5	0.85	0.48	-	-
Immunoglobulin G2, μg/mL	-	-	25.2	28.3	0.94	0.09	-	-

^1^The data are expressed as the mean ± SEM, *n* = 15. ^a,b,c^*P* < 0.05

^2^*DSCC* Differential somatic cell count, *– 1 Wk* Week – 1 relative to calving, + *1 Wk* Week + 1 relative to calving

^3^*Hs* Health status, *Wk* Sampling week, *Hs* × *Wk* Interaction between health status and sampling week

### Prognostic potential of prepartum blood NETs for postpartum mastitis risk

**Fig. 4:**
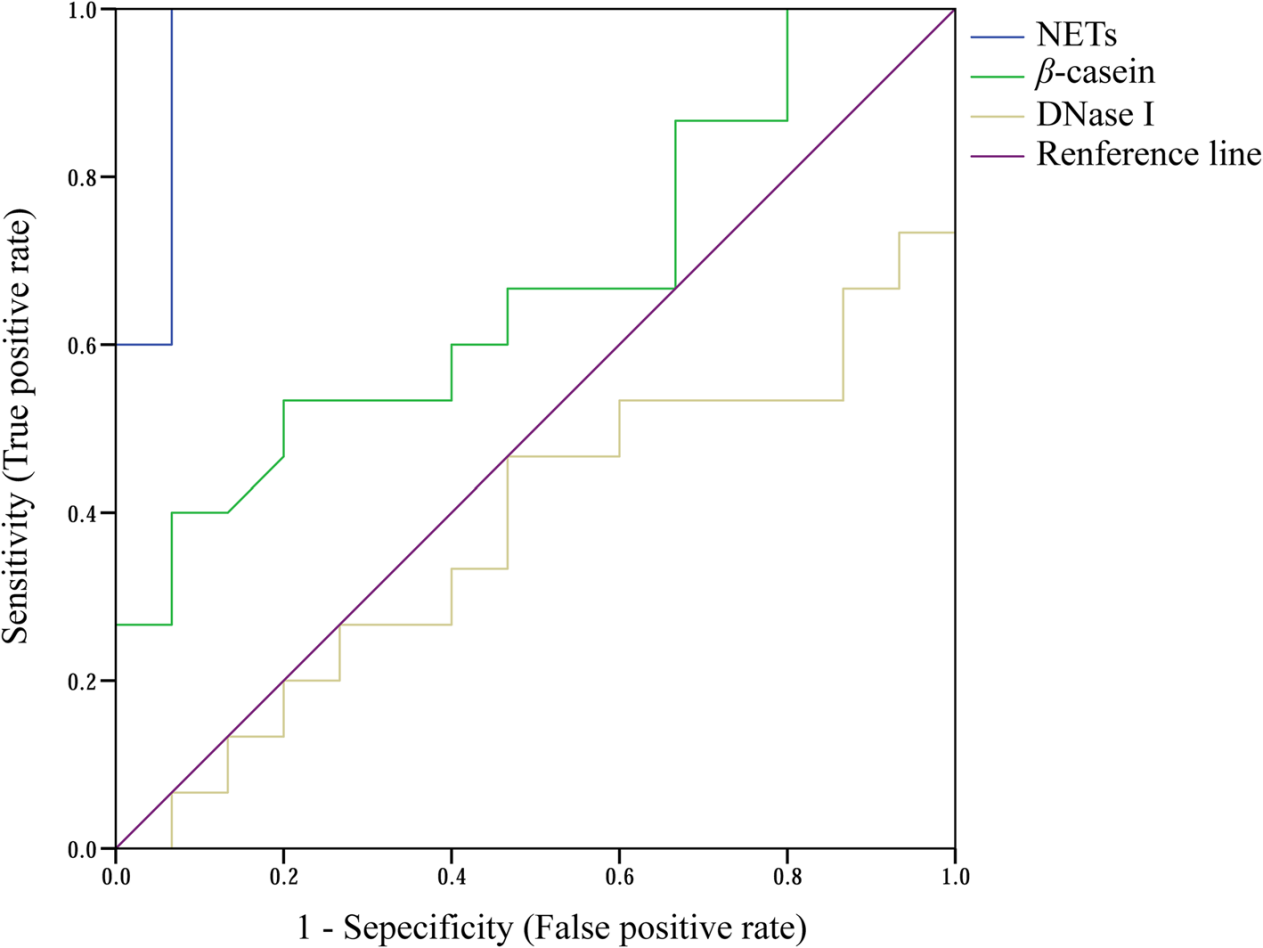
Receiver operator characteristic curves of serum variables in dairy cows with low- and high-differential somatic cell counts at week – 1 relative to calving. *n* = 15. NETs: Neutrophil extracellular traps; DNase I: Deoxyribonuclease I

The ROC curves for serum NETs, DNase I, and *β*-casein in dairy cows with low and high DSCC are shown in Fig. 4 and Table 5. Among the assessments of three parameters (NETs, DNase I, and *β*-casein), the highest AUC (0.973) was observed for outstanding diagnosis using NETs. There were no discriminations observed (AUC < 0.7) for the DNase I and *β*-casein in prepartum serum. In addition, only using NETs level resulted in a *P* value lower than 0.05, and the critical value for NETs levels was 32.2 ng/mL with high sensitivity (1.00) and specificity (0.93). No significant *P* values were observed (*P* > 0.05) in DNase I and *β*-casein of prepartum dairy cows.

**Table 5:** Receiver operator characteristic analysis of serum indices in dairy cows with low- and high-differential somatic cell counts at week – 1 relative to calving^a^

Items^b^	Variables^c^					
	AUC	SEM	*P*-value	Sensitivity	Specificity	Critical value
NETs	0.973	0.028	< 0.01	1.00	0.93	32.2
DNase I	0.618	0.105	0.27	0.53	-0.33	84.1
*β*-casein	0.669	0.100	0.12	0.53	0.33	5.4

^a^*n* = 15

^b^*NETs* Neutrophil extracellular traps, *DNase I* Deoxyribonuclease I

^c^*AUC* Area under the curve, *SEM* Standard errors of the mean

## Discussion

Dairy cows are at high risk for mastitis during the transition period, which can be effectively characterized by either SCC or DSCC [6, 19], sometimes by combination of DSCC and SCC values [20]. In the current study, we focused on the potential prognosis of the health of transition cows based on blood neutrophil extracellular traps (NETs). Thus, we considered that the DSCC, expressed as the percentage of neutrophils plus lymphocytes in total somatic cells, could be better reflective of the mammary health of dairy cows than SCC. In fact, the change in SCC and DSCC showed a similar tendency in the current study. Therefore, DSCC but not SCC is used in this study. In the present study, DSCC in postpartum milk was mainly associated with NETs concentrations in serum and milk, which supported the opinion that the formation of blood NETs in transition dairy cows could increase the risk of postpartum mastitis [12]. Although much work has been done between mammary immunobiology and the control of mastitis [21], more work is warranted on the relationship among the formation of NETs, mastitis risk, and their diagnostic potential.

The transition period is the most critical for the health of dairy cows, and the risk for bovine mastitis is high from week – 1 to + 1 relative to calving [22, 23]. The stability of hematological cell number and function is important in protecting the host organism from immune assault [24]. In addition, serum nutrient metabolite homeostasis reflects the state of the health of transition dairy cows [25]. In the current study, the higher levels of red blood cells, hemoglobin, and hematocrit and lower concentrations of cholesterol and albumin in cows with higher DSCC suggested that dairy cows with high mastitis risk may be prone to diseases and have higher cell permeability due to protein mobilization [26, 27]. In agreement with our results, several studies showed that the levels of red blood cells, cholesterol, and albumin in dairy cows prone to disease changed [28, 29]. However, the numbers of other blood cells, especially neutrophils, and nutritional metabolite levels in transition dairy cows with different DSCCs were almost the same, indicating that these cows with different DSCCs had similar physiological statuses that were consistent with the lack of clinical signs. More work is needed to investigate the internal correlation between neutrophil function and cow health.

Regarding health status, oxidative stress occurs as an imbalance between ROS generation and endogenous antioxidation [30]. Thus, excessive ROS concentrations are crucial in initiating oxidative stress [31]. In general, oxidative stress is enhanced in the mammary gland of dairy cows with diseases such as mastitis and ketosis [32, 33]. In this study, dairy cows with high mastitis risk exhibited higher levels of oxidative stress. The 8-OHdG represents the degree of DNA peroxidation [31]. NETs, which comprise the decondensed chromatin and granule contents (such as MPO and NE) of neutrophils, rely on ROS production and are accompanied by cell DNA damage [34, 35]. DNase I is reported to be beneficial to animal health through promoting the clearance of NETs caused by neutrophil aggregation [36]. For the cows in the present study, serum ROS might have stimulated neutrophils to release NETs from blood into the mammary gland in the dairy cows with high mastitis risk. From the perspective of individual differences, the serum levels of ROS and NETs were always higher in dairy cows with high mastitis risk from prepartum to postpartum in this work. Therefore, the blood NETs with different DSCCs might be a potential indicator of mastitis risk from late gestation to early lactation. However, the predictive power of NETs still needs more work. The NETosis is beneficial to dairy cow health, but the excessive generation of NETs may result in lipid and protein peroxidation of the tissues, which may be indicated by the activities of MDA and PC, respectively [31]. In the current study, dairy cows with high mastitis risk presented higher levels of oxidative damage to lipids and proteins. Recent studies also supported that the formation of NETs in cows with disease can increase oxidative damage to the body [37]. The Formation of NET was involved in mammary epithelial cell damage in vitro [38]. Therefore, no difference was found in ROS levels of low DSCC dairy cows between prepartum and postpartum, but the ROS levels were enhanced postpartum in dairy cows with high DSCC, suggesting that NETs could damage the blood-milk barrier integrity in transition cows with high mastitis risk. In addition, our previous results of the correlation between the level of milk NETs and serum lactose level after calving suggest a possible association between NETosis and the breakdown of blood-milk barrier integrity. The causal relationship among NETs, blood-milk barrier, and DSCC still needs to be further investigated in vivo and in vitro.

The intact blood-milk barrier inhibits a disordered exchange of soluble and cellular components between blood and milk in the mammary gland. Structural damage to the blood-milk barrier is accompanied by its increased permeability [39]. Lactose, *α*-lactalbumin, and *β*-casein are synthesized exclusively by mammary epithelial cells and can only be transferred from milk to blood via a paracellular pathway through disrupted tight junctions. Thus, their levels in blood can be used as indicators of the disruption of blood-milk barrier integrity [14, 40]. Similarly, the Na^+^/K^+^ ratio in milk is known to be inversely correlated with blood-milk barrier integrity [40]. In addition, levels of blood-derived proteins, such as BSA, IgG1, and IgG2, increase in milk when the blood-milk barrier is leaky [14]. Among the blood-derived proteins, IgG2 has a larger size (150 kDa) than those of *β*-casein (24 kDa), *α*-lactalbumin (14 kDa), and BSA (66.5 kDa). These proteins are thought to reach the destination through passive paracellular transport, but IgG1 is the active transcellular transport protein [41]. Wall et al. reported that the blood-milk barrier permeability is high in general during the early lactation period [14]. In the current study, the cows with high mastitis risk showed higher levels of larger proteins (*β*-casein in blood and IgG2 in milk) passing through blood-milk barrier, suggesting that blood-milk barrier is nonselective for indicators with smaller molecular weights or active transcellular transport proteins during the transition period, but higher permeability of blood-milk barrier exists in cows with higher DSCC. Consistent with the trend of blood NETs, the β-casein concentrations in serum tended to be higher in dairy cows with high mastitis risk throughout the transition period. However, from the perspective of physiology in transition cows, the contributions of animal factors such as hormones need to be further investigated. Taken together, these findings suggest that the different levels of blood NETs in transition dairy cows with low and high DSCC may affect the permeability of the blood-milk barrier, and could be acted as potential indicators of mastitis risk in dairy cows during the transition period.

The prognostication of mastitis is an integral component of transition dairy cow management, and mastitis biomarkers are required for effective diagnosis and prognosis [42]. Our previous work showed that the ratio of neutrophils to platelets could be prognostic for mastitis [43], but the underlying mechanism was not clear. In this study, the results of the assessments of hematological cells and nutrient metabolites in serum confirmed that postpartum dairy cows are more susceptible to diseases than prepartum cows. Oxidative damage-related parameters, the formation of blood NETs, and blood-milk barrier permeability in dairy cows with high mastitis risk exist throughout the transition period. The ROC curves suggest that NETs are more related to mastitis and more prognostically predictive for it than other markers, which is consistent with the correlation of the DSCC ratios and level of serum NETs in prepartum cows. The critical value of prepartum serum NETs is 32.2 ng/mL, providing prognostic value to determine the occurrence of mastitis during the transition period. However, for mastitis diagnosis, we also need the pathological perspective in the future to verify the diagnostic potential of NETs combined with the intramammary infection assessment and clinical symptoms. In brief, NETs are formed in dairy cows before calving, which may cause the breakdown of the blood-milk barrier and result in blood immune cells entering milk through disrupted tight junctions. This could exist throughout the transition period and may explain the variations of milk DSCC.

## Conclusion

In this study, dairy cows with high milk DSCC showed changes in the levels of hematological parameters and nutrient metabolites, higher levels of serum oxidative species and NETs formation, and higher permeability of the blood-milk barrier than those of the cows with low DSCC. Our results indicated that the formation of NETs in transition dairy cows may disrupt the blood-milk barrier and thereby increase the mastitis risk in postpartum dairy cows. We propose that prepartum NETs in the blood may act as a novel target for the prediction of the mammary health risk in transition dairy cows.

## Data Availability

All data generated or analyzed during this study are included in this published article.

## Abbreviations

ALT: Alanine aminotransferase
AST: Aspartate aminotransferase
AUC: Area under the curve
BHBA: β-Hydroxybutyrate
BSA: Bovine serum albumin
DCFH-DA: Dichlorofluorescein-diacetate
DNase I: Deoxyribonuclease I
DSCC: Differential somatic cell count
Hs: Health status
IgG: Immunoglobulin G
MDA: Malondialdehyde
MPO: Myeloperoxidase
NE: Neutrophil elastase
NEFA: Nonesterified fatty acid
NETs: Neutrophil extracellular traps
PC: Protein carbonyls
ROC: Receiver operator characteristic
ROS: Reactive oxygen species
SCC: Somatic cell count
Wk: Sampling week
8-OHdG: 8-Hydroxy-2-deoxyguanosine
